# *Koliella bifissiva* sp. nov (Chlorellaceae, Chlorophyta) and Analysis of Its Organelle Genomes

**DOI:** 10.3390/plants13182604

**Published:** 2024-09-18

**Authors:** Huiyin Song, Hai Peng, Zhiwei Fang, Baolong Zhang, Zhaolu Zhu, Zilan Xiao, Guoxiang Liu, Yuxin Hu

**Affiliations:** 1 Institute for Systems Biology, School of Life Sciences, Jianghan University, Wuhan 430056, China; songhy@jhun.edu.cn (H.S.);; 2 Key Laboratory of Algal Biology, Institute of Hydrobiology, Chinese Academy of Sciences, Wuhan 430072, China; liugx@ihb.ac.cn; 3 Changjiang Basin Ecology and Environment Monitoring and Scientific Research Center, Changjiang Basin Ecology and Environment Administration, Ministry of Ecology and Environment, Wuhan 430010, China

**Keywords:** Chlorellaceae, *Koliella*, taxonomy, chloroplast genome, mitochondrial genome

## Abstract

Chlorellacean members are common in aquatic or subaerial habitats, and many of them have significant economic value. Taxonomic reports and organelle genome data for the *Nannochloris* clade, an important subgroup within this family, are limited, hindering the understanding and exploitation of this clade. In this study, a fusiform-celled strain, FACHB-3607, was isolated from a pond in China. Through examination of morphological characteristics and phylogenetic analyses of rbcL, 18S rDNA, and ITS, it was identified as a new species within the *Nannochloris* clade, named *Koliella bifissiva* sp. nov. In addition, this study provided a first insight into the organellar genomes of the genus *Koliella*. The *K. bifissiva* chloroplast had a 99.8 kb genome, and the mitochondrion had a 40.8 kb genome, which are moderate sizes within the *Nannochloris* clade. Phylogenomic analysis showed that *K. bifissiva* is most closely related to *Nannochloris* sp. “desiccata”, followed by *Marvania*. In contrast, *Picochlorum* was the most distantly related species. The organelle genomes of the *Nannochloris* clade display dynamic evolution, reflected in variations in genome size, gene content and order, and selection pressure. This research enhances our knowledge of species diversity and evolutionary history in the *Nannochloris* clade.

## 1. Introduction

The members of the family Chlorellaceae (Trebouxiophyceae, Chlorophyta) display diverse morphological characteristics [1,2] and are found in various environments such as water, wet soil, or bark. Some members of this family, such as *Chlorella* Beyerinck [Beijerinck] and *Auxenochlorella* (Shihira & Krauss) Kalina & Puncochárová, have desirable traits. They are high in nutritional value, rich in bioactive substances, have rapid metabolism, and hold important economic value in food, medicine, health products, energy, and agriculture [3]. The Chlorellaceae consist of the *Chlorella* clade, the *Parachlorella* clade, the *Nannochloris* clade, the AHP clade, and some clades consisting of a single genus [2,4]. Most research on this family focuses on small coccoid algae with the *Chlorella* clade and the *Parachlorella* clade, and there are few reports on fusiform-celled members in the *Nannochloris* clade. The *Nannochloris* clade, an important subgroup within this family, is found in freshwater, saline water, and moist soil. Members of this clade reproduce in various ways, such as binary fission (*Nannochloris* Naumann, *Koliella* Hindák, *Gloeotila contorta* (Lemmermann) Chodat, *Catena viridis* Chodat), budding (*Marvania* Hindák), and autosporulation (*Picochlorum* Henley et al.) [5,6,7]. The diversified reproductive methods set them apart from other clades in the family Chlorellaceae. Notably, the pyrenoid is absent in all known members of the *Nannochloris* clade. Some members within the *Nannochloris* clade exhibit distinctive physical characteristics. *Pumiliosphaera acidophila* is acidophilic and was isolated from an acidic volcano stream and soil nearby (pH 2.0) [8]. *Chloroparva pannonica* was isolated from a turbid, shallow soda pan [9]. *Nannochloris* sp. “desiccata” (formerly *Chlorella desiccata*) and *Picochlorum* show excellent characteristics, including rapid growth rate, high productivity, halophilic nature, and potential for algal production in marine environments. They also offer promising genetic tools for developing strains suitable for expressing transgenes inserted into the nuclear or chloroplast genomes [10,11,12]. Their fast growth and high biomass yield render them ideal for large-scale cultivation and commercial exploitation. This makes them particularly suitable for aquaculture and biotechnology applications. However, due to the uncommon nature of its members, there are few reports and taxonomic studies on this group. The fusiform-celled *Koliella*, cylindrical-celled *Gloeotila contorta*, and *Catena viridis* have only been sporadically reported internationally [6,13], and have not yet been reported in China. The discovery of new species, especially those are rare or infrequently reported, has great significance for biodiversity and related research.

The genus *Koliella* was established based on morphological differences from the genus *Raphidonema* Lagerh [14]. While *Raphidonema spiculiforme* Vischer was a solitary organism, cells of other *Raphidonema* species formed short filaments. Due to these distinct morphological characteristics, *R. spiculiforme* was transferred out of *Raphidonema,* and the genus *Koliella* was established. The type species of *Koliella* is *Koliella spiculiformis* (Vischer) Hindák, which was previously known as *Raphidonema spiculiforme*. The authentic strain CCAP 470/2A (equivalent to UTEX 340) of *R. spiculiformis*, was isolated from a freshwater habitat in Graubünden, Switzerland by Vischer in 1940, and is currently preserved in the Culture Collection of Algae and Protozoa (https://www.ccap.ac.uk/, accessed on 16 July 2024). *Koliella* was initially classified within the order Ulotrichales [14]. Subsequently, several species were described on the basis of various morphological characteristics, such as the length-width ratio and cell dimensions. These include *Koliella elongata* (Nygaard) Nygaard [15], *Koliella setiformis* Nygaard [16], and *Koliella budapestinensis* Hortobágyi [17], followed by *Koliella sigmoidea* Hindák, *Koliella spirulinoides* Hindák, *Koliella norvegica* Hindák, and *Koliella crassa* Hindák [18]. Further additions to the genus were made with the descriptions of *Koliella spiralis* Kuosa by Kuosa in 1988 [19], *Koliella variabilis* (Nygaard) Hindák, *Koliella bratislaviensis* (Hindák) Hindák, and *Koliella antarctica* Andreoli et al. [20]. In 1996, Hindák established a new family, Koliellaceae, to accommodate *Koliella* and its related genera [21]. Until then, the taxonomy of this genus had primarily relied on morphological studies. *Koliella* contains both freshwater and snow-borne algal species, characterized by spindle-shaped unicells. Katana et al. (2001) revealed for the first time the phylogenetic position of genus *Koliella* based on partial 18S rDNA and 16S rDNA, and the genus appeared to be polyphyletic [22]. Surprisingly, the type species, *K. spiculiformis*, was proved to belong to Chlorellaceae (Chlorophyta, Trebouxiophyceae). More recently, Yakimovich et al. (2021) pointed out the authentic strain of the type species, *K. spiculiformis* CCAP 470/2A. Their analysis using rbcL genes and ITS regions revealed that *Koliella* forms a clade that is a sister to *Chlorella* spp. within the family Chlorellaceae [13]. However, it is unfortunate that authentic strains of most species within this genus have been lost, which poses a challenge for further taxonomic and phylogenetic studies.

The analysis of organelle genomes is important for understanding phylogenetic relationships, evolutionary history, and taxonomy. Currently, there is limited data on the organelle genome of the *Nannochloris* clade compared to related clades such as the *Chlorella*, *Parachlorella*, and AHP clades. To date, the NCBI database contains eight chloroplast genomes (cpDNAs) and seven mitochondrial genomes (mtDNAs) retrieved from three genera (*Marvania*, *Nannochloris*, and *Picochlorum*) [11,23,24]. There has been no comparative analysis of organelle genomes of the *Nannochloris* clade. This hinders our understanding of the characteristics of the organelle genomes in the *Nannochloris* clade.

In this study, we isolated the strain FACHB-3607 from a pond located in Hubei Province, China. After analyzing its morphological characteristics and molecular markers, we identified it as a new species of the genus *Koliella*. This discovery marks the first report of the genus *Koliella* in China. Additionally, we constructed its complete chloroplast genome and mitochondrial genome, which represent the first chloroplast and mitochondrial genomes of the genus *Koliella*. Furthermore, we conducted a comparative genomic analysis among species within the *Nannochloris* clade.

## 2. Results

### 2.1. Morphological Observation and Taxonomic Implication

**Figure 1:**
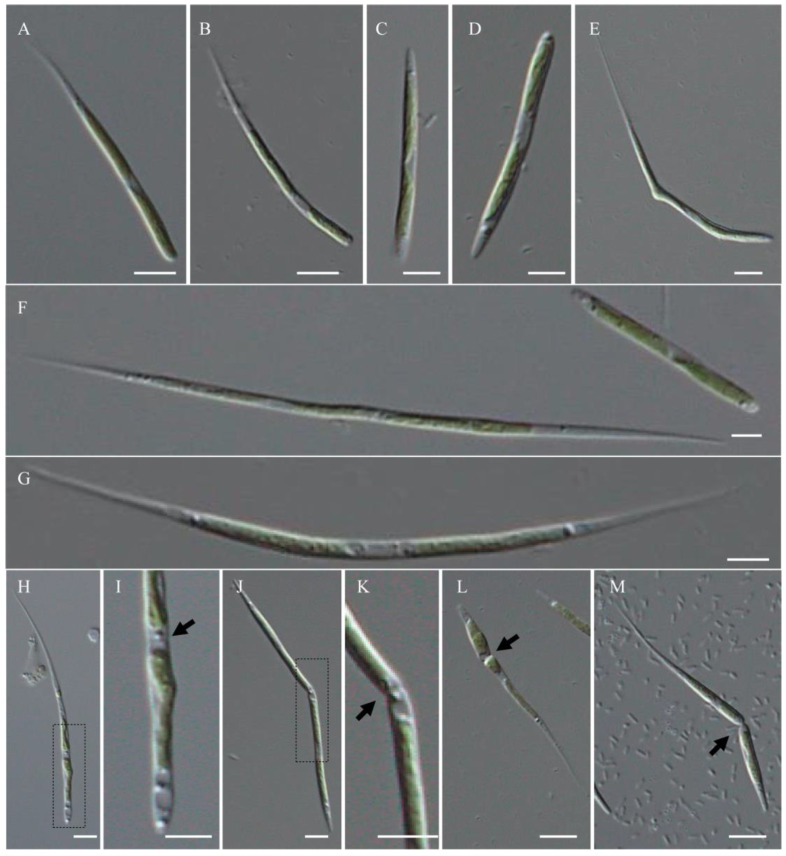
Microphotographs of *Koliella bifissiva* sp. nov. strain FACHB-3607. Scale bars = 5 μm. (**A**,**B**) Cells with one apex obtuse and the other apex long and bristle-like; (**C**,**D**) cells with two blunt ends or one end slightly tapered; (**E**) cell with an embossment; (**F**) solitary mature cell with two long bristle-like points, and solitary young cell with two rotund points; (**G**) solitary mature cell with two long bristle-like points, cell crescent; (**H**) cells that were dividing and the daughter cell where the wall has formed; (**I**) amplification of the black-dashed rectangular box in (**H**); (**J**) 2-celled colony; (**K**) amplification of the black-dashed rectangular box in (**J**); (**L**,**M**) 2-celled colonies containing newly formed daughter cells. The black arrow indicates where the cell divides.

*Koliella bifissiva* H. Song, Y. Hu, and G. Liu sp. nov. (Figure 1).
Description

Planktonic. Solitary, fusiform, or strongly elongate, curved, or straight, with obtuse or rotund apices, or tapering gradually into (very) long bristle-like points. Chloroplast single and parietal, without pyrenoid. Because the chloroplast does not extend into the ends, the apices are hyaline. The cell length was 6–64 times the cell width and 1.5–2.4 μm in width and 8.5–96 μm in length. Cells without mucilage. Asexual reproduction by binary fission. *K. bifissiva* differed from other *Koliella* species by its distinct rbcL, ITS, and 18S rDNA sequences.

Holotype: Material from the authentic strain FACHB-3607 was deposited in the Freshwater Algae Culture Collection at the Institute of Hydrobiology (FACHB collection, https://algae.ihb.ac.cn), China, in 5 July 2024.

Type locality: a pond in Wuhan, Hubei Province, China (114°4′22″, 30°35′6″).

Habitat: freshwater, planktonic.

Etymology: the species was named for its binary reproduction.

Authentic strain: strain FACHB-3607.

Live microscopy

*K. bifissiva* was planktonic and found in a freshwater pond in China. Its cells were mostly solitary, fusiform, filiform, or elongate. Most of the cells were asymmetrically curved, and a few were straight. The apices were radially tapering into long bristle-like points, or obtuse, or rotund (Figure 1), and were hyaline because the chloroplast does not extend into the ends. The young cells tended to be shorter and often appeared round at one or both ends (Figure 1A–D). Mature cells tended to be longer and were more likely to have bristle-like points at one or both ends (Figure 1E–G). The range of cell length was quite large, from 15 μm to 96 μm, while the middle width of the cell was relatively stable, ranging from 1.5 μm to 2.4 μm. The cell length was 6–64 times the cell width. The chloroplast was parietal and had a missing lobe where the nucleus is, and no pyrenoid was observed. The cell wall was thin, hyaline, and without mucilage. Some cells had an embossment, which may be where the cell is going to divide. Asexual reproduction occurred by cell division into two asymmetrical or equal parts (Figure 1H–K). Two-celled colonies (Figure 1L,M), which is a state where cells have just division but have not completely separated, were rare.

### 2.2. Phylogenetic Analyses

**Figure 2:**
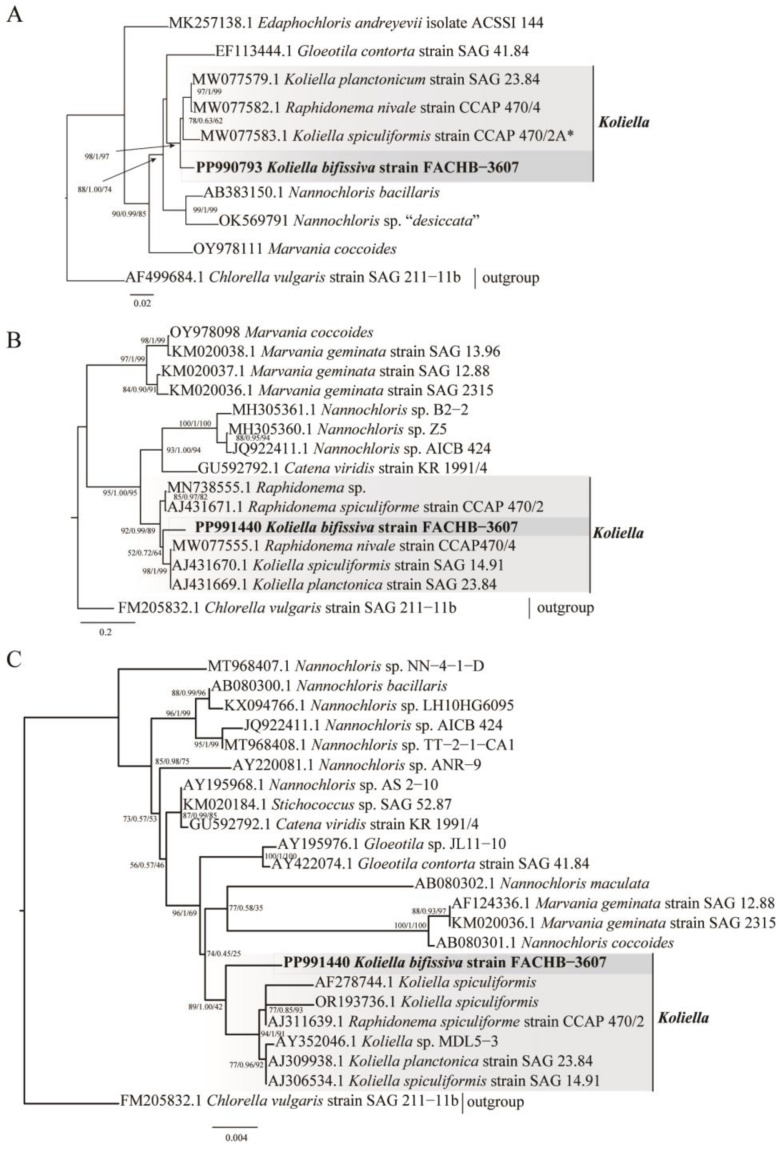
Phylogenetic analysis of strain FACHB-3607 and related species. The Sequences for phylogenetic analysis were selected based on a BLAST search (5 July 2024), and all reliable *Koliella* sequences were added to the phylogenetic analysis. The phylogenetic analysis was performed using the maximum likelihood method and a Bayesian (BI) method with IQ-TREE multicore version 2.0.3. *Chlorella vulgaris* was used as an outgroup. (**A**) Phylogenetic analysis based on rbcL genes. The analysis involved 10 rbcl sequences, with a total of 1330 positions in the final dataset. The substitution model was TIM3e + I + G4, which was selected based on the Bayesian Information Criterion (BIC). The asterisk marks the authentic strain. (**B**) Phylogenetic analysis based on ITS sequences. The analysis comprised 15 sequences, with a total of 755 positions in the final dataset. The substitution model used was TIM2 + F + G4, chosen according to the BIC using IQ-TREE multicore version 2.0. (**C**) Phylogenetic analysis based on 18S rDNA sequences. The analysis comprised 20 sequences, with a total of 1709 positions in the final dataset. The substitution model used was TNe + I, chosen according to the BIC using IQ-TREE multicore version 2.0.3.

The phylogeny was reconstructed using the rbcL dataset (Figure 2A), the ITS dataset (Figure 2B), and the 18S rDNA dataset (Figure 2C). Based on rbcL-based phylogenetic analyses, strain FACHB-3607 was identified with strong support as an individual branch sister to the clade that included *K. spiculiformis* strain CCAP 470/2A, *Raphidonema nivale* strain CCAP 470/4, and *Koliella planctonicum* strain SAG 23.84. Strain CCAP 470/2A was the authentic strain of *K. spiculiformis*. Yakimovich et al. (2021) [13] and our genetic analyses revealed that strain CCAP 470/4, formerly known as *Raphidonema nivale*, does not belong to *Raphidonema* and was a member of this *Koliella* genus. The phylogenetic analyses based on ITS showed strain FACHB-3607 was nested within the genus *Koliella* and formed an individual branch and was different from all known species. The 18S rDNA of strain FACHB-3607 was very long because of the presence of five introns with a combined length of 2135 bp. Based on intron-removed 18S rDNA phylogenetic analyses, strain FACHB-3607 was identified as an individual branch sister to other species of the genus *Koliella*. In conclusion, the phylogenetic analyses using multiple genetic markers positioned strain FACHB-3607 within the genus *Koliella*, and was different from the known species.

### 2.3. Characteristics of the Chloroplast Genome

**Figure 3:**
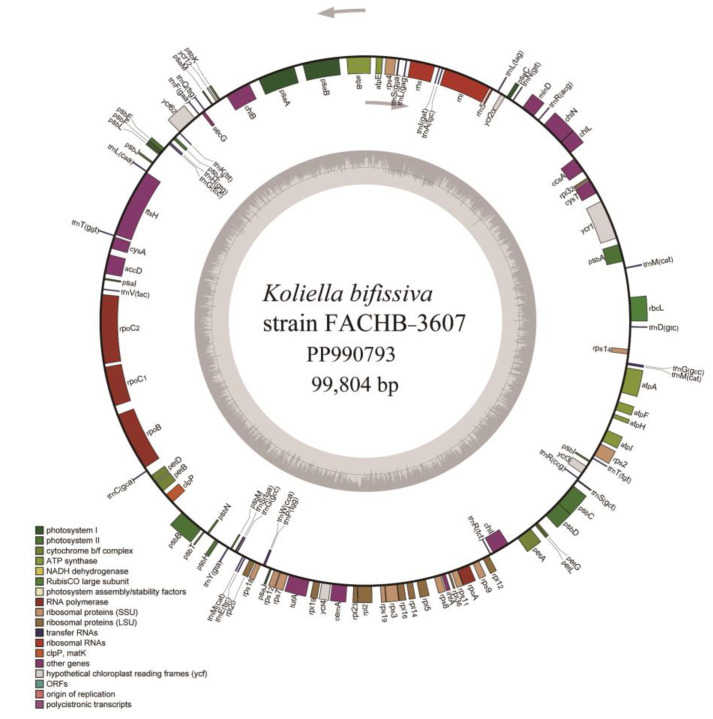
Circular map of the chloroplast genome of *Koliella bifissiva* sp. nov. strain FACHB-3607. The transcription of genes is done clockwise for genes inside the circle and counterclockwise for genes outside of it. Different colors correspond to different functional groupings’ genes. The inner circle’s dark and light gray hues represent the AT and GC contents, respectively.

We assembled the complete circular cpDNA of strain FACHB-3607, which was 99,804 bp in size. This cpDNA of strain FACHB-3607 lacked inverted repeat regions (IRa, IRb) as shown in Figure 3, consistent with other species in the *Nannochloris* clade. The GC content was 32.8%, which was slightly higher than that of *Picochlorum* (32–32.2%), slightly lower than that of *Nannochloris* sp. “desiccata” (33.4%), and significantly lower than that of *Marvania* (37.6–38.2%) within the *Nannochloris* clade. Annotation of the cpDNA revealed that it included 79 protein-coding genes (PCGs), 31 tRNA genes, and three rRNA genes, without introns.

**Figure 4:**
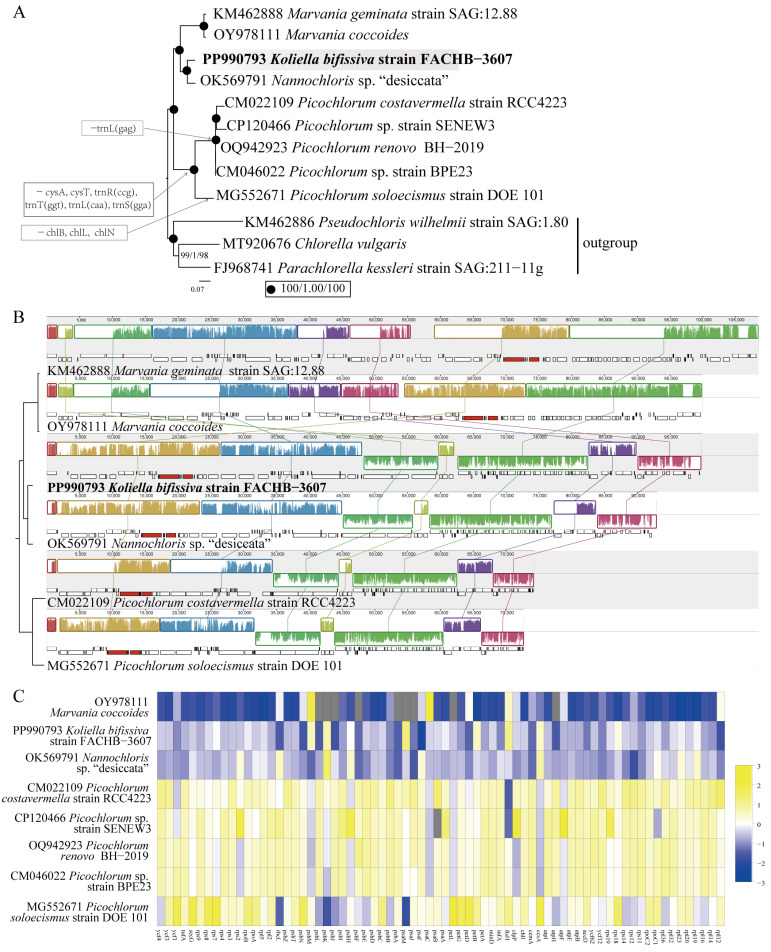
Chloroplast genome analysis of *Koliella bifissiva* sp. nov. strain FACHB-3607. (**A**) Phylogenetic tree of the *Nannochloris* clade based on 79 concatenated proteins of chloroplast genomes. *Parachlorella kessleri* (FJ968741), *Chlorella vulgaris* (MT920676), and *Pseudochloris wilhelmii* (KM462886) were used as outgroups. The phylogenetic tree was reconstructed utilizing IQ-TREE, with the optimal model calculated for each gene separately. The numbers displayed on the branches indicate SH-aLRT support (%)\a Bayesian support\ultrafast bootstrap support (%), respectively, with our strains highlighted in bold. Evolutionary scenarios of gene losses in the *Nannochloris* clade are labeled in the box. (**B**) Synteny comparison of six cpDNAs of the *Nannochloris* clade using Mauve. Collinear portions of sequences are shown by rectangular blocks of the same hue. Collinear blocks with vertical bars within them demonstrate a degree of sequence identity. (**C**) The Ka/Ks values of chloroplast genes within the *Nannochloris* clade and *Marvania geminata* were used as reference. The data were standardized (column scale) to facilitate comparison of different taxa. The gray rectangular box indicates that Ka/Ks values are absent.

A phylogenomic analysis of the *Nannochloris* clade based on 79 concatenated proteins of all nine known cpDNAs was conducted (Figure 4A). The results revealed that *K. bifissiva* strain FACHB-3607 and *Nannochloris* sp. “desiccata” are clustered together and are closely related, while *M. coccoides* and *M. geminata* cluster together and are closely related. The branch containing *K. bifissiva* strain FACHB-3607 and *Nannochloris* sp. “desiccata” and the branch containing *M. coccoides* and *M. geminata* form sister groups with robust support. The four cpDNAs mentioned above share 79 protein-coding genes (PCGs), 31 tRNA genes, and three rRNA genes. *Picochlorum* was found at the base of the *Nannochloris* clade and lost some cpDNA genes. All known *Picochlorum* strains lacked cysA, cysT, trnR(ccg), trnT(ggt), trnL(caa), and trnS(gga) genes compared to *K. bifissiva*, *Nannochloris* sp. “desiccata”, and *Marvania*. Additionally, chlB, chlL, and chlN were lost in *Picochlorum soloecismus*, and trnL(gag) was lacking in the lineage consisting of four *Picochlorum* strains. The cpDNAs of the *Nannochloris* clade might be undergoing reductive evolution.

Additionally, a synteny analysis of cpDNAs of strain FACHB-3607 and related species was performed. A comparison of genome organization and gene order is shown in Figure 4B. Ten conserved gene blocks were identified among the six cpDNAs. *K. bifissiva* strain FACHB-3607, *Nannochloris* sp. “desiccata”, and more distant *Picochlorum* showed nearly identical gene orders, with some minor differences. First, compared to *K. bifissiva* strain FACHB-3607, rps14 and trnD(gtc) were reversed in *Nannochloris* sp. “desiccata”. Second, compared to *K. bifissiva* strain FACHB-3607, trnG(tcc) and trnH(gtg) were reversed in *Picochlorum*.

The selection pressure was analyzed based on the chloroplast genes, and the cpDNA of *Marvania geminata* was used as a reference. The results showed that the Ka/Ks values of all chloroplast protein-coding genes were less than 1, indicating that these genes were generally affected by purification selection (Figure 4C).

### 2.4. Characteristics of the Mitochondrial Genome

**Figure 5:**
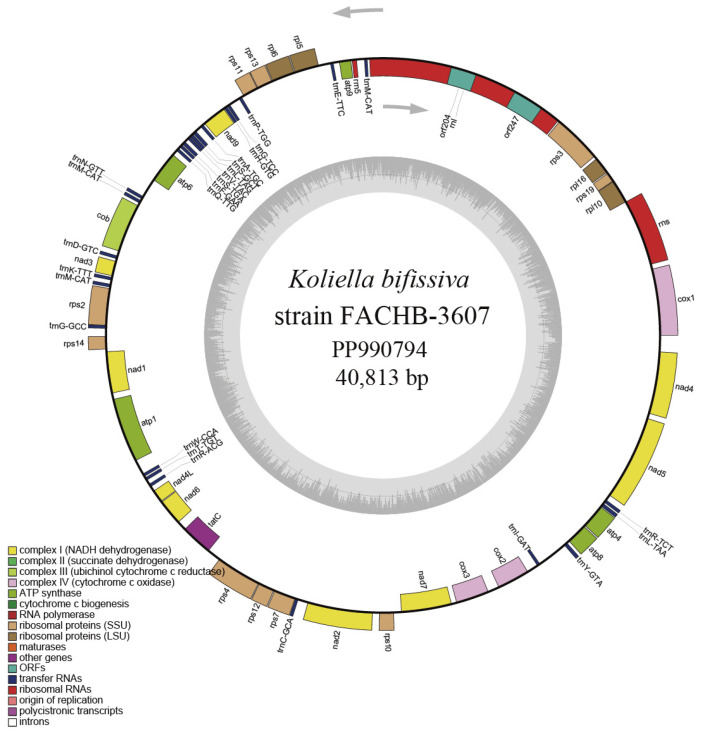
Circular map of the mtDNA of *Koliella bifissiva* sp. nov. strain FACHB-3607. The transcription of genes is done clockwise for genes inside the circle and counterclockwise for genes outside of it. Different colors correspond to different functional groupings’ genes. The inner circle’s dark and light gray hues represent the AT and GC contents, respectively.

We also successfully assembled the complete circular mtDNA of strain FACHB-3607, and the size was 40,813 bp (Figure 5). The GC content was 29.8%, which was slightly higher than that of *Marvania* (29.2–29.4%), slightly lower than that of *Nannochloris* sp. “desiccata” (32.2%), and significantly lower than that of *Picochlorum* (40.9–42.3%). The mtDNA of strain FACHB-3607 contained 33 PCGs, 26 tRNA genes, and three rRNA genes. The gene distribution was compact, and the total length of the gene sequence accounted for 86% of the genome, which was similar to *Nannochloris* sp. “desiccata” (85%), higher than *Marvania coccoides* (71%), and *Marvania geminata* (66%), and lower than most *Picochlorum* spp. (83–96%). Introns were found in the rnl gene; introns were also found in the rnl genes of *Nannochloris* sp. “desiccata” and *Marvania*, but were absent in *Picochlorum* spp.

**Figure 6:**
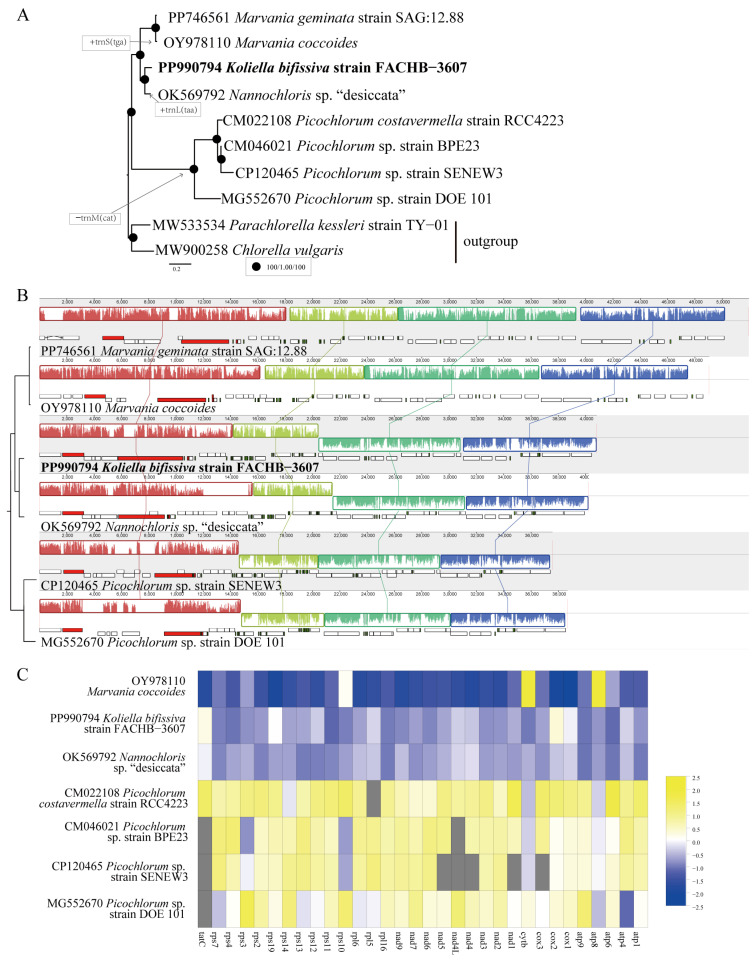
Mitochondrial genome (mtDNA) analysis of *Koliella bifissiva* sp. nov. strain FACHB-3607. (**A**) Phylogenetic tree of the *Nannochloris* clade based on 33 concatenated proteins of mtDNAs. *Chlorella vulgaris* (MW900258), *Parachlorella kessleri* (MW533534), and *Pseudochlorella signiensis* (PP746560) were used as outgroups. The phylogenetic tree was reconstructed utilizing IQ-TREE, with the optimal model calculated for each gene separately. The numbers displayed on the branches indicate SH-aLRT support (%)\a Bayesian support\ultrafast bootstrap support (%), respectively, with our strains highlighted in bold. Evolutionary scenarios of gene losses (−) and acquisition (+) in the *Nannochloris* clade are labeled in the box. (**B**) Synteny comparison of six mtDNAs of the *Nannochloris* clade using Mauve. Collinear portions of sequences are shown by rectangular blocks of the same hue. Collinear blocks with vertical bars within them demonstrate a degree of sequence identity. (**C**) The Ka/Ks values of mitochondrial genes within the *Nannochloris* clade and *Marvania geminata* were used as reference. The data were standardized (column scale) to facilitate comparison of different taxa. The gray rectangular box indicates that Ka/Ks values are absent.

We then conducted a phylogenomic analysis of the *Nannochloris* clade based on 33 shared proteins of all known mtDNAs (Figure 6A). The results revealed the same phylogenomic topology as the cpDNA-based phylogenomic tree (Figure 4A). *K. bifissiva* strain FACHB-3607 and *Nannochloris* sp. “desiccata” form a sister group with strong support, and *M. coccoides* and *M. geminata* form a sister group with robust support. The clade containing *K. bifissiva* strain FACHB-3607 and *Nannochloris* sp. “desiccata” and the clade containing *M. coccoides* and *M. geminata* form sister groups with robust support. The genus *Picochlorum* was sistered to all other members of the *Nannochloris* clade. The mtDNAs of the *Nannochloris* clade share 33 protein-coding genes (PCGs), 26 tRNA genes, and three rRNA genes, except *Nannochloris* sp. “desiccata”, which contains an extra gene, trnL(caa). All strains of *Picochlorum* have lost one copy of trnM(cat), and *M. coccoides* contains an extra gene, trnS(tga).

We also conducted a synteny analysis of mtDNAs of strain FACHB-3607 and related species, and the comparison of genome organization and gene order is shown in Figure 6B. Four conserved gene blocks were identified among the six DNAs. *K. bifissiva* strain FACHB-3607 and *Nannochloris* sp. “desiccata” showed identical gene orders, while *Marvania* exhibited two inverted and reversed blocks and *Picochlorum* exhibited one inverted and reversed block compared to *K. bifissiva* strain FACHB-3607.

This paper also analyzed selection pressure in the mitochondrial genome, using *M. geminata*’s mitochondrial genome as a reference (Figure 6C). The results show that most mitochondrial protein-coding genes experienced purifying selection, with Ka/Ks ratios all less than 1, except for the *atp8* gene of *M. coccoides*, whose Ka/Ks ratio was >1. The *atp8* gene of *M. coccoides* showed clear positive selection characteristics and indicated ongoing positive selection.

### 2.5. Sequence Variations between K. bifissiva Strain FACHB-3607 and Nannochloris sp. “desiccata”

**Figure 7:**
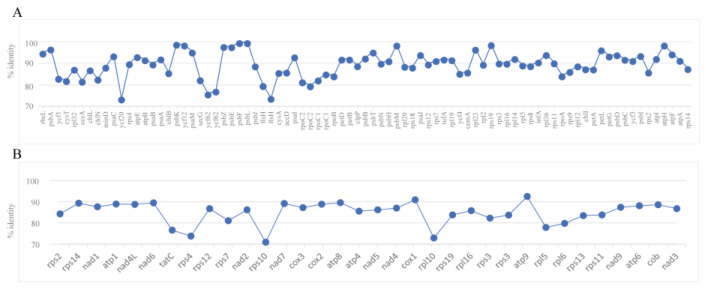
The percent identification (sequence similarity) of protein-coding genes of *Nannochloris* sp. “desiccata” compared to *K. bifissiva* strain FACHB-3607. The genes are shown in the order in which they appear in the genome. (**A**) The percent identification (sequence similarity) of 79 protein-coding genes of chloroplast genomes; (**B**) the percent identification (sequence similarity) of 33 protein-coding genes of mitochondrial genomes.

*K. bifissiva* strain FACHB-3607 and *Nannochloris* sp. “desiccata” showed the closest relative based on either mitochondrial or chloroplast genome analysis. Therefore, this study compared *K. bifissiva* strain FACHB-3607 and *Nannochloris* sp. “*desiccata*” in both their mitochondrial and chloroplast genomes to explore the differences in the protein-coding gene sequences between the two species. The results, shown in Figure 7, revealed that the sequence similarity between the two species for 79 shared genes in the chloroplast genome ranged from 73% (*ycf20*) to 99% (*psbF*), with an average similarity of 89%. Notably, only 68% of gene *ycf62* (679 bp of 1164 bp) and 78% of gene *rpoC2* (3199 bp of 4095 bp) could be aligned, indicating significant differences in these two genes between the two species. In the mitochondrial genomes, genetic similarity ranged from 71% (*rps10* gene) to 93% (*atp9* gene), with an average similarity of 84%. Furthermore, 43% (678 bp of 1188 bp) of gene rps3 failed to be aligned, further indicating substantial differences in the rps3 gene in the mitochondrial genome between the two species. Overall, the sequences of the organelle genomes in the two strains were very different, both in the chloroplast genome and the mitochondrial genome, and the mitochondrial genome showed a slightly lower degree of variation compared to the chloroplast genome.

## 3. Discussion

The morphological characteristics of strain FACHB-3607 unquestionably fit with the description of *Koliella* [14]: The cells are usually solitary, usiform, filiform, or (strongly) elongate. They can be straight or curved, with obtuse or rotund apices or gradually tapering into (very) long bristle-like points. After division, cells usually detach immediately. The cell membrane is thin and hyaline, without mucilage. It contains a single parietal chloroplast without a pyrenoid. The organism reproduces asexually by binary fission.

Based on the information from Algaebase, *Koliella* consists of 21 accepted species names. Although most of these species have limited descriptions, we still find that the morphological characteristics of strain FACHB-3607 differ from those of other species. For instance, while the daughter cells of *Koliella closterioides* (Kufferath) Hindák, *Koliella crassa* Hindák, *Koliella helvetica* (Kol) Hindák, *Koliella pyrenoidifera* (Korshikov) Hindák, and *K. spirulinoides* remain joined together for a relatively long time after division [18,25], the daughter cells of strain FACHB-3607 detach immediately. Additionally, *K. sigmoidea*, *K. spiralis*, and *Koliella tatrae* (Kol) Hindák have sigmoid cells, while strain FACHB-3607 does not [21]. The *Koliella viretii* (Chodat) Hindák cells are wider than those of strain FACHB-3607, and the *Koliella longiseta* (Vischer) Hindák and *Koliella spirotaenia* (West) Hindák cells are longer. Furthermore, the *K. norvegica* cells have bifurcated ends at each pole, while strain FACHB-3607 cells do not. The *Koliella variabilis* cells are strongly or irregularly curved, while strain FACHB-3607 cells are not. Unfortunately, certain species, such as *Koliella alpina* (Kol) Hindák, *Koliella bernina* (Kol) Hindák, *K. bratislaviensis*, *Koliella chodatii* (Kol) Hindák, *Koliella nivalis* Hindák, and *K. setiformis* were only described at the time of their first report, and no detailed information is searchable. Furthermore, the geographical distribution of these species differs significantly from that of strain FACHB-3607, which is found in China. *K. spiculiformis*, which was first isolated in Switzerland and was related to strain FACHB-3607 based on phylogenetic analysis (Figure 2), is likewise geographically distinct from strain FACHB-3607.

Molecular analyses were crucial to identify microalgae in the family Chlorellaceae, leading to the discovery of *Chloroparva*, *Planktochlorella*, *Laetitia sardoa*, and others [9,26,27].In this study, phylogenetic analysis using multiple molecular markers revealed that strain FACHB-3607 differs from all known *Koliella* species and forms an independent clade nested with *Koliella*. It is worth mentioning that the sequences in the GenBank database named *Koliella longiseta*, *Koliella corcontica*, and *Koliella sempervirens* were mislabeled or need further taxonomic revisions and were actually *Raphidonema* members within the Prasiolales, which are far from the genus *Koliella*. Combined with morphological characteristics, phylogenetic results, and geographical distribution characteristics, strain FACHB-3607 represents a new species.

The study also offers the first insight into the organellar genomes of *Koliella* and a comparative analysis of the organelle genome of the *Nannochloris* clade. The results show that the *K. bifissiva* strain FACHB-3607 has moderately sized and moderately compact organelle genomes. *K. bifissiva* is most closely related to *Nannochloris* sp. “desiccata”, followed by *Marvania*. In contrast, *Picochlorum* is the most distantly related species. This kind of relationship is supported by phylogenomic analysis and selection pressure analysis.

In the selection pressure analysis between species, consistent trends were obtained based on mitochondrial genomes and chloroplast genomes. When compared to *M. geminata*, *Marvania coccoides* has the lowest Ka/Ks ratio, indicating that it has evolved under comparable selection pressures to *M. geminata*. Additionally, there was little difference in the Ka/Ks ratios of *Nannochloris* sp. “desiccata” and *Koliella*, suggesting that similar selection pressures were probably acting on both species. On the other hand, compared to *Marvania*, *Koliella*, and *Nannochloris* sp. “desiccata”, the Ka/Ks ratios of most of the genes in *Picochlorum* were significantly higher. This indicates that *Picochlorum* was subjected to more selection pressure, leading to adaptive changes in its gene sequence.

The *Nannochloris* clade displays dynamic evolution, reflected in variations in genome size, gene content and order, and selection pressure. The differences in genome size might be explained by gene, intron, and/or intergenic region reduction events. Notably, specific cpDNA genes such as *cysA*, *cysT*, trnR(ccg), trnT(ggt), trnL(caa), and trnS(gga) were lost in the common ancestor of *Picochlorum*. Furthermore, *chlB*, *chlL*, and *chlN* were further lost in the *P. soloecismus* strain DOE 101 (MG552671) and one copy of trnL(gag) was lost in the common ancestor of the lineage formed by four other *Picochlorum* species. The functions of *cysA* and *cysT* are linked and related to the sulfate ABC transporter [28], which normally either coexist or are absent in plastids of the Chlorellaceae. In contrast, the *chlB*, *chlL*, and *chlN* genes are associated with chlorophyll synthesis in the dark. These genes were lost in the *Pedinomonas minor* [29] and *Pseudendoclonium akinetum* [30] of Chlorophyta, suggesting possible evolutionary patterns. However, the mitochondrial gene content appears to be highly conserved among the analyzed species.

Additionally, the study shows that genome rearrangements sometimes occur between closely related species, and gene order sometimes remains consistent between distantly related groups, indicating the need for caution when interpreting the effects of genome rearrangement on evolution due to limitations in reflecting species’ affinities. Undoubtedly, genomic rearrangements are the driving force of genetic diversity, profoundly affecting gene expression and protein function, which maybe in turn shapes the environmental adaptability of species.

This research helps us better understand the diversity of species and provides new insights into the evolutionary history of the *Nannochloris* clade. It is worth noting that there were significant sequence variations between the *K. bifissiva* strain FACHB-3607 and *Nannochloris* sp. “desiccata”, yet these two are grouped together in the phylogenomic analysis. This might be due to a lack of organelle genome sequencing of taxa. It will be necessary for future research to expand the range of organelle genome sequencing of taxa in the *Nannochloris* clade.

## 4. Materials and Methods

### 4.1. Strain Isolation and Culture

The strain FACHB-3607 under study was isolated from a water sample collected on 14 October 2023 (the weather was cloudy) from a fish pond (<0.05 km^2^, no algal blooms occurred, open water) in Wuhan, Hubei Province, China (114°4′22″, 30°35′6″). Isolation technique: a single cell was isolated using a micropipette under an inverted microscope and transferred to a 96-well cell culture plate with BG11 fluid medium. After expanding the culture, the strain was transferred to a 25 cm^2^ cell culture flask. Culture conditions: the strain was cultured in BG11 fluid medium at 25 °C with a light intensity of 30 μmol m^−2^ on a 12:12 h light–dark cycle. The strain FACHB-3607 was deposited in the Freshwater Algae Culture Collection at the Institute of Hydrobiology (FACHB collection), Wuhan, Hubei Province, China.

### 4.2. Light Microscopy

Live cells in the log phase were observed using a ZEISS Axio Imager Z2 light microscope (Zeiss, Sliedrecht, The Netherlands) with a ×100 oil immersion objective. Images were captured with a Zeiss Axiocam 506 color digital camera.

### 4.3. DNA Extraction and Whole Genome Sequencing

Total nucleic acids were extracted using the DNAsecure Plant Kit (Tiangen Biotech, Beijing, China). DNA quality was assessed on 1% agarose gels. The electrophoresis bands were clear, with the main band being over 15 kb. DNA concentration was measured with the Qubit^®^ DNA Assay Kit on a Qubit^®^ 3.0 Fluorometer (Invitrogen, Waltham, MA, USA). The sequencing library was prepared using the NEB Next^®^ Ultra™ DNA Library Prep Kit for Illumina (NEB, Ipswich, MA, USA) following the manufacturer’s instructions, with index codes for each sample. Genomic DNA was sonicated to 350 bp. Then, DNA fragments were end polished, A-tailed, and ligated with the full-length adapter for Illumina sequencing, followed by further PCR amplification. PCR products were purified with the AMPure XP system (Beckman Coulter, Beverly Hills, CA, USA), and DNA concentration was quantified with the Qubit^®^ 3.0 Fluorometer. Libraries were size-analyzed by NGS3K/Caliper and quantified by real-time PCR (3 nM). Index-coded samples were clustered using the cBot Cluster Generation System with the Illumina PE Cluster Kit (Illumina, San Diego, CA, USA). The DNA libraries were sequenced on the Illumina platform (Novaseq-PE150), generating 150 bp paired-end reads.

### 4.4. Quality Control, Assembly, and Annotation

Quality control was conducted as previously outlined in Song et al. (2021) [31]. The 18S rDNA and ITS sequences were assembled using GetOrganelle [32] with the following parameter settings: “10-k 21, 45, 65, 85, 105-F embplant_nr”, utilizing publicly available 18S rDNA + ITS sequences of *Chlorella sorokiniana* (AB731602.1), *Didymogenes sphaerica* (AB731603.1), and *Didymogenes soliella* (AB731605.1) as references. The cpDNA was assembled using GetOrganelle with the following parameter settings: “15-k 21, 45, 65, 85, 105-F embplant_pt” referencing the cpDNAs of *Chlorella vulgaris* (MK948102.1), *Micractinium pusillum* (MN649872.1), *Parachlorella kessleri* (NC_012978.1), *Dicloster acuatus* (NC_025546.1), and *Chlorella heliozoae* (NC_036805.1). The mtDNA was assembled using GetOrganelle with the following parameter settings: “embplant_mt-R 50-k 21, 45, 65, 85, 105 -P 1,000,000” referencing the mtDNAs of *Chlorella vulgaris* (MW900258), *Micractinium variabile* (MT332838), *Micractinium singularis* (MN894286), and *Micractinium pusillum* (MN649871). The sequences were validated by aligning sequencing reads to the assembled sequences using BWA Version 0.7.17-r1188 [33] and SAMtools Version: 1.7 [34], and visualized with IGV version 2.17.0 [35]. The cpDNA and mtDNA were annotated using MFannot (https://megasun.bch.umontreal.ca/apps/mfannot/, accessed on 18 July 2024) and BLAST homology searches. The ribosomal RNAs (rRNAs) were identified with barrnap v0.9 (https://github.com/tseemann/barrnap, accessed on 18 July 2024) and manually verified. The annotated data have been submitted to the GenBank databases with accession numbers PP991440 (18S rDNA, ITS), PP990793 (chloroplast genome), and PP990794 (mitochondrial genome). The introns in the 18S rDNA were annotated using sequence alignment with other closely related species. The intronic ORFs of organelle genomes were annotated using Mfannot.

### 4.5. Phylogenetic Inference

The strain FACHB-3607 was identified based on the phylogenetic analysis of rbcL, 18S rDNA, and ITS sequences. Sequences for phylogenetic analysis were selected based on a BLAST search conducted on 5 July 2024, and the authentic strain of *K. spiculiformis* (CCAP 470/2A) was included and all selected sequences were downloaded from GenBank (http://www.ncbi.nlm.nih.gov). The alignment of sequences was performed using MAFFT v7.471 [36], with manual adjustments made to ambiguously aligned regions using MEGA7 [37]. The alignments were uploaded as Supplementary Data. The phylogenetic analysis based on rbcL sequences (Figure 1A) comprised 10 sequences, with a total of 1330 positions in the final dataset. The substitution model used was GTR + F + I + G4, chosen according to BIC using IQ-TREE multicore version 2.0.3 [38]. The phylogenetic analysis based on ITS sequences (Figure 1B) included 15 sequences, with a total of 755 positions in the final dataset. The substitution model used was TIM2 + F + G4, chosen according to BIC using IQ-TREE multicore version 2.0.3. The phylogenetic analysis based on 18S rDNA sequences (Figure 1C) comprised 20 sequences, with a total of 1709 positions in the final dataset. The substitution model used was TNe + I, chosen according to BIC using IQ-TREE multicore version 2.0.3. Phylogenetic analysis was conducted using the maximum likelihood method and the Bayesian inference (BI) method with IQ-TREE multicore version 2.0.3.

### 4.6. Phylogenomic Analysis

The cpDNA-based phylogenomic tree was constructed using all nine known cpDNAs of the *Nannochloris* clade. Data preparation for the analysis was performed using PhyloSuite v1.2.2 [39]. The 79 shared proteins (RPOC1, YCF1, RPL2, RPOC2, RPS2, RPS3, YCF3, RPS4, YCF4, RPL5, RPS7, RPS8, RPS9, RPS11, RPL12, RPS12, YCF12, RPL14, RPS14, RPL16, RPS18, RPL19, RPS19, RPL20, YCF20, RPL23, RPL32, RPL36, YCF62, ACCD, ATPA, ATPB, ATPE, ATPF, ATPH, ATPI, CCSA, CEMA, CHLB, CHLI, CHLL, CHLN, CLPP, CYSA, CYST, FTSH, INFA, MIND, PETA, PETB, PETD, PETG, PETL, PSAA, PSAB, PSAC, PSAI, PSAJ, PSAM, PSBA, PSBB, PSBC, PSBD, PSBE, PSBF, PSBH, PSBI, PSBJ, PSBK, PSBL, PSBM, PSBN, PSBT, PSBZ, RBCL, RPOA, RPOB, SECG, and TUFA) were extracted using PhyloSuite v1.2.2, and were aligned with MAFFT v7.471 using the G-INS-i (accurate) strategy. TrimAl v1.2 [40] was used to eliminate poorly matched sites (including the intronic ORFs) in the alignments through a heuristic method (-automated1). And then, the 79 proteins were concatenated; the final dataset consisted of 18,947 sites. The best-fit partition model for each gene was determined by the BIC criterion. The phylogenetic analysis was performed using the maximum likelihood method and a Bayesian (BI) method with IQ-TREE multicore version 2.0.

All eight of the *Nannochloris* clade’s known mtDNAs were used to construct the mtDNA-based phylogenomic tree. PhyloSuite v1.2.2 was used to prepare the data for analysis. A total of 33 proteins (ATP1, COX1, NAD1, COX2, NAD2, RPS2, COX3, NAD3, RPS3, ATP4, NAD4, RPS4, NAD5, RPL5, ATP6, NAD6, RPL6, NAD7, RPS7, ATP8, ATP9, NAD9, RPL10, RPS10, RPS11, RPS12, RPS13, RPS14, RPL16, RPS19, CYTB, NAD4L, AND TATC) were aligned with MAFFT v7.471 employing the G-INS-i (accurate) strategy. TrimAl v1.2 was utilized to eliminate poorly matched sites in the alignments through a heuristic method (-automated1). The best-fit partition model for each gene was determined by the BIC criterion. The 33 proteins were concatenated, and the final dataset consisted of 8055 sites. The phylogenomic analysis was performed using the maximum likelihood method and a Bayesian (BI) method with IQ-TREE multicore version 2.0.

### 4.7. Synteny Analysis and Selective Pressure Analysis

The synteny comparison was visualized using mauve ver. 2.3.1 under the progressive mode [41]. The ratio of nonsynonymous substitution (Ka) to synonymous substitution (Ka), Ka/Ks, was used to measure the natural selection acting on the proteins. This value was calculated with TBtools-II [42] and a heatmap was also drawn with TBtools.

## 5. Conclusions

In this study, a new species, *Koliella bifissiva* sp. nov., was described based on phylogenetic location and morphological characteristics. Additionally, the first chloroplast genome and mitochondrial genome of the genus *Koliella* were reported, and its genomic characteristics were analyzed. Comparative genomic analysis revealed that the organelle genomes of the *Nannochloris* clade display dynamic evolution, reflected in variations in genome size, gene content and order, and selection pressure. This study increases our understanding of species diversity and provides valuable insights into the genomic characteristics of the *Nannochloris* clade. In the future, we will focus on the genetic diversity, geographical distribution, and physiological and biochemical characteristics of *Koliella bifissiva* to enhance our understanding of the species’ ecological adaptability and evolutionary potential.

## Data Availability

The data generated in this article are available in the GenBank databases (https://www.ncbi.nlm.nih.gov/) with accession numbers PP991440, PP990793, and PP990794.

## Supplementary Materials

The following supporting information can be downloaded at: https://www.mdpi.com/article/10.3390/plants13182604/s1, the *gene alignment data of* Figure 2A–C.
